# Leadless pacemaker implantation in real‐world clinical practice: An Italian survey promoted by the AIAC (Italian Association of Arrhythmology and Cardiac Pacing)

**DOI:** 10.1002/joa3.70045

**Published:** 2025-03-24

**Authors:** Roberto Rordorf, Valentina De Regibus, Luca Bontempi, Guido De Ambroggi, Giosuè Mascioli, Patrizio Mazzone, Matteo Anselmino, Michela Casella, Maurilio Lauretti, Gemma Pelargonio, Vincenzo Russo, Manola Vilotta, Matteo Ziacchi, Giuseppe Boriani, Pietro Palmisano, Sakis Themistoclakis, Antonio D'Onofrio, Roberto De Ponti, the AIAC Ricerca Investigator Network

**Affiliations:** ^1^ Fondazione IRCCS Policlinico S. Matteo Arrhythmia and Electrophysiology Unit, Division of Cardiology Pavia Italy; ^2^ Cardiology Division, Department of Cardio‐Thoracic‐Vascular Diseases ASST Santi Paolo Carlo Milan Italy; ^3^ Division of Cardiology ASST Bergamo Est, Bolognini Hospital Seriate Italy; ^4^ Arrhythmology and Electrophysiology Centre IRCCS Multimedica Sesto San Giovanni Italy; ^5^ Division of Cardiology ASST del Garda, Desenzano del Garda Hospital Desenzano del Garda Italy; ^6^ Cardiology 3, Electrophysiology Niguarda Hospital Milan Italy; ^7^ Cardiology Division, “Città Della Salute e Della Scienza di Torino” Hospital, Department of Medical Sciences University of Turin Torino Italy; ^8^ Cardiology and Arrhythmology Clinic University Hospital “Azienda Ospedaliero‐Universitaria Delle Marche” Ancona Italy; ^9^ Department of Clinical, Special and Dental Sciences Marche Polytechnic University Ancona Italy; ^10^ UOSVD Elettrofisiologia Ospedale Vito Fazzi Lecce Italy; ^11^ Institute of Cardiology Catholic University of Sacred Heart Rome Italy; ^12^ Department of Cardiovascular Sciences Fondazione Policlinico Universitario Agostino Gemelli IRCCS Rome Italy; ^13^ Cardiology and Syncope Unit, Department of Translational Medical Sciences, Monaldi Hospital University of Campania Luigi Vanvitelli Naples Italy; ^14^ Department of Medicine and Surgery University of Insubria Varese Italy; ^15^ Cardiology Unit, Ospedale di Circolo ASST Settelaghi Varese Italy; ^16^ Cardiology Unit, Dipartimento Cardio‐Toraco‐Vascolare IRCCS Azienda Ospedaliero‐Universitaria di Bologna Bologna Italy; ^17^ Cardiology Division, Department of Biomedical, Metabolic and Neural Sciences University of Modena and Reggio Emilia, Policlinico di Modena Modena Italy; ^18^ Cardiology Unit “Card. G. Panico” Hospital Tricase Lecce Italy; ^19^ Cardiology Division Ospedale dell'Angelo Mestre Venice Italy; ^20^ Departmental Unit of Electrophysiology, Evaluation and Treatment of Arrhythmias Monaldi Hospital Naples Italy

**Keywords:** leadless pacemaker, pacemaker implantation, survey

## Abstract

**Backgrounds:**

After a decade since the introduction of leadless pacemaker (L‐PM), its use is still limited. The aim of this survey is to evaluate how this technology is perceived by electrophysiologist members of a National scientific society in clinical practice.

**Methods:**

A questionnaire with 22 questions was posted in the reserved area of the society website. The multiple‐choice questions concerned the center's characteristics, patient selection criteria, limitations to the L‐PM use, implant procedures, and follow‐up. Additionally, non‐implanting centers were also allowed to participate by completing the initial nine questions.

**Results:**

Ninety‐two responders participated in this survey: 59% implanted <20 L‐PM yearly and 31% did not implant L‐PM. The three main reasons to choose an L‐PM were anatomic contraindications to a transvenous pacemaker, the patient's high infective risk, and previous lead extraction, accounting for 78%, 74%, and 64% of the responses, respectively. Age >60 years was indicated as more suitable by most of the responders. Among the implanting centers, the main limitation to a wider adoption was cost (49%), the lack of atrial pacing (28%), the absence of a dedicated extraction tool, and data on replacement (22%). The L‐PM implant was performed with only local anesthesia in 77% of the centers and was associated with limited procedure duration and fluoroscopy time even in low‐volume centers.

**Conclusions:**

Although the L‐PM implant is not a particularly complex procedure, these data confirm that its use is currently limited to selected patients of older age. Cost decreases and new developments might increase the adoption of this technology.

## Graphical abstract

A survey of Italian electrophysiologists identified key reasons for choosing L‐PM: anatomic contraindications, high infection risk, and prior lead extraction. Barriers include cost, lack of atrial pacing, and extraction tools. The use of L‐PM implantation is currently limited to selected patients of older age. Wider adoption may follow cost reduction and technological advances.

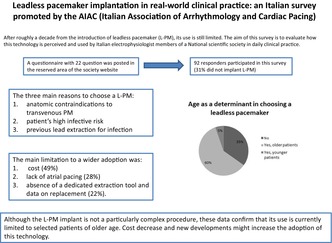

## 1 INTRODUCTION

Transvenous lead pacemaker (PM) has been, for many decades, and still is, the cornerstone treatment of bradyarrhythmias. Nevertheless, conventional transvenous PM therapy is burdened by a not negligible rate of complications, ranging from around 9% to 12% according to the most recent observational studies.^1^, ^2^, ^3^, ^4^ It is also well known that device‐related complications significantly increase health‐related costs.^5^, ^6^ In order to overcome complications related to endovascular leads and subcutaneous pockets, leadless PM (L‐PM) has been developed and introduced into clinical practice.^7^, ^8^, ^9^ L‐PM has been proven to be as effective and safe as traditional transvenous pacemakers.^10^, ^11^, ^12^ Current guidelines indicate that L‐PMs should be considered when no upper extremity venous access is feasible or when the risk of device pocket infection is particularly high. In other situations, the implantation of an L‐PM might be considered on a case‐by‐case basis, taking into consideration life expectancy and using shared decision‐making.^13^ However, there are still some issues that significantly limit a wider use of L‐PM in everyday clinical practice, confining this therapy to a niche of patients. Higher costs, the unsettled issue on how to manage generator replacement, and the limited availability and poor experience with dual‐chamber L‐PM^14^, ^15^ are among the factors affecting the wider use of L‐PMs in real‐world practice. Moreover, how L‐PMs are currently managed from patients' selection, procedure workflow, and follow‐up is still poorly described. Accordingly, the Italian Association of Arrhythmology and Cardiac Pacing (AIAC) decided to promote this survey to better portray how this newer technology is perceived and utilized among Italian centers.

## 2 METHODS

From February 2021 to April 2023, a survey promoted by the AIAC was published on its official website (https://www.aiac.it/). This survey was open to physician members of the association operating in Italian centers, both implanting and non‐implanting L‐PM. Participation in the survey was voluntary. In general, one physician from each center could complete the questionnaire, although multiple responses from high‐volume centers were accepted as multiple implanting teams may be present.

The questionnaire consisted of 22 questions: five focused on the characteristics of the participating center (i.e., annual volume of PM implants, the distribution of implants by PM type), four on the criteria for choosing a leadless over a transvenous PM, one on the limitations or barriers to L‐PM implantation, seven on organizational aspects of L‐PM implantation, and five on follow‐up. All questions were multiple‐choice, and two allowed multiple responses. The first nine questions of this survey could be completed also by physicians operating in centers not implanting L‐PMs. The full text of this survey is available in the Supplementary Materials. Data were analyzed as aggregate and, when deemed appropriate, sub‐analyzed according to the center volume.

### 2.1 Statistical analysis

Descriptive statistics were reported as means for normally distributed continuous variables. Continuous variables with skewed distribution were reported as medians with 25–75th percentiles. Categorical data were expressed as percentages, reported in contingency tables, and compared by means of χ2 test or Fisher's exact test, as appropriate. *p*‐values <0.05 were considered statistically significant.

## 3 RESULTS

### 3.1 Participating centers

**FIGURE 1:**
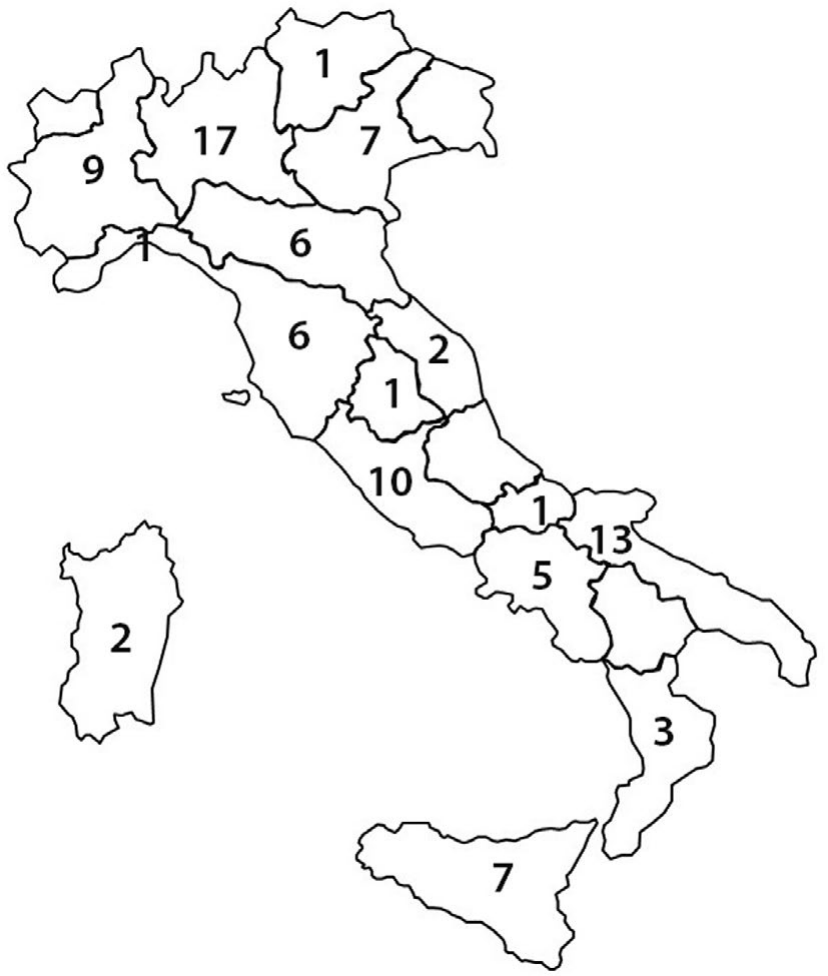
Regional distribution of the responders.

Overall, 92 responders from 81 centers completed the survey. The list of the participants is reported in the Appendix A. The participating centers accounted for 44.5% of the 182 Italian centers performing both catheter ablation and all types of cardiac implantable electronic device implants for treatment of cardiac arrhythmias.^16^ The participating centers were geographically distributed as reported in Figure 1: a median of five responders per region was observed (range: 0–17; interquartile range: 1–7), and, in nine regions, five or more physicians participated. The number of participating centers compared with the total number of operating centers was similar in Northern, Central, and Southern Italy (29%, 33%, 28%, *p* = 0.32).

**FIGURE 2:**
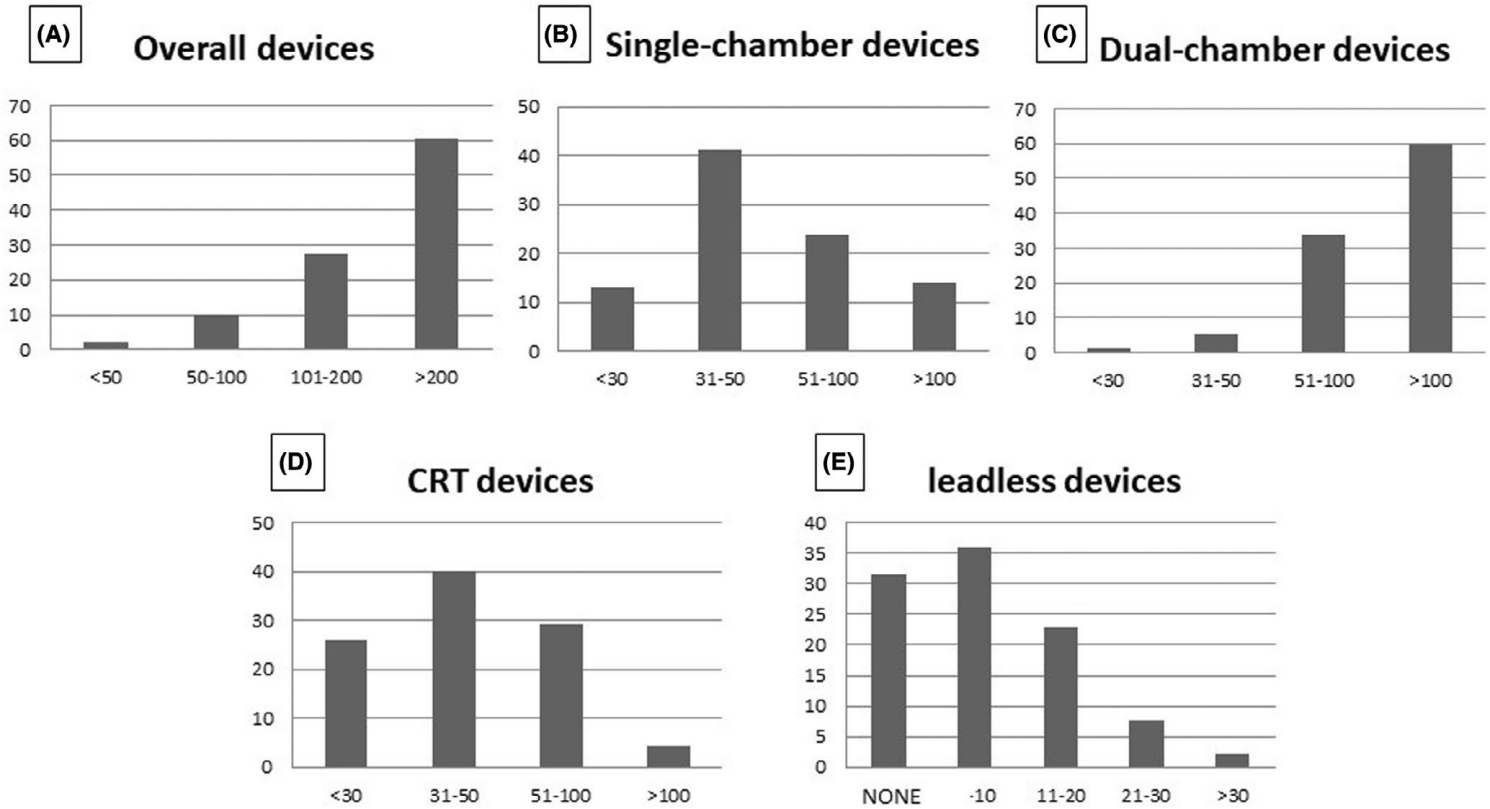
Volume of new annual device implants in the participating centers: Overall (A), single‐chamber (B), dual‐chamber (C) pacemakers, cardiac resynchronization therapy devices (D), and leadless pacemakers (E). The percentage of centers is reported on the vertical axis, while the range of implanted devices is on the horizontal axis. CRT, cardiac resynchronization therapy.

As shown in Figure 2, most of the participating centers (60%) had a high volume of de novo implants of any type of PMs (Figure 2A) and of dual‐chamber PMs (Figure 2C) per year. Forty percent of the centers had an intermediate volume (31–50) of new implants of single‐chamber PMs (Figure 2B) and devices for cardiac resynchronization therapy (Figure 2D) per year. Interestingly, as shown in Figure 2E, 29 responders (31%) had never implanted a L‐PM, whereas the majority implanted up to 10 (36%) or between 11 and 20 (23%) L‐PMs per year; only a minority of the centers implanted between 21 and 30 or more than 30 L‐PMs per year (8% and 2%, respectively).

### 3.2 Reasons for choosing a leadless versus a transvenous pacemaker

When the participants were asked to state their three main reasons to prefer implanting a L‐PM instead of a transvenous PM, anatomic contraindication to a transvenous PM was chosen by 78% of the responders, followed by the patient's high infective risk (74%) and previous transvenous PM extraction for infection (64%). Six participants preferred not to answer, as they claimed to not have enough experience in L‐PM.

**FIGURE 3:**
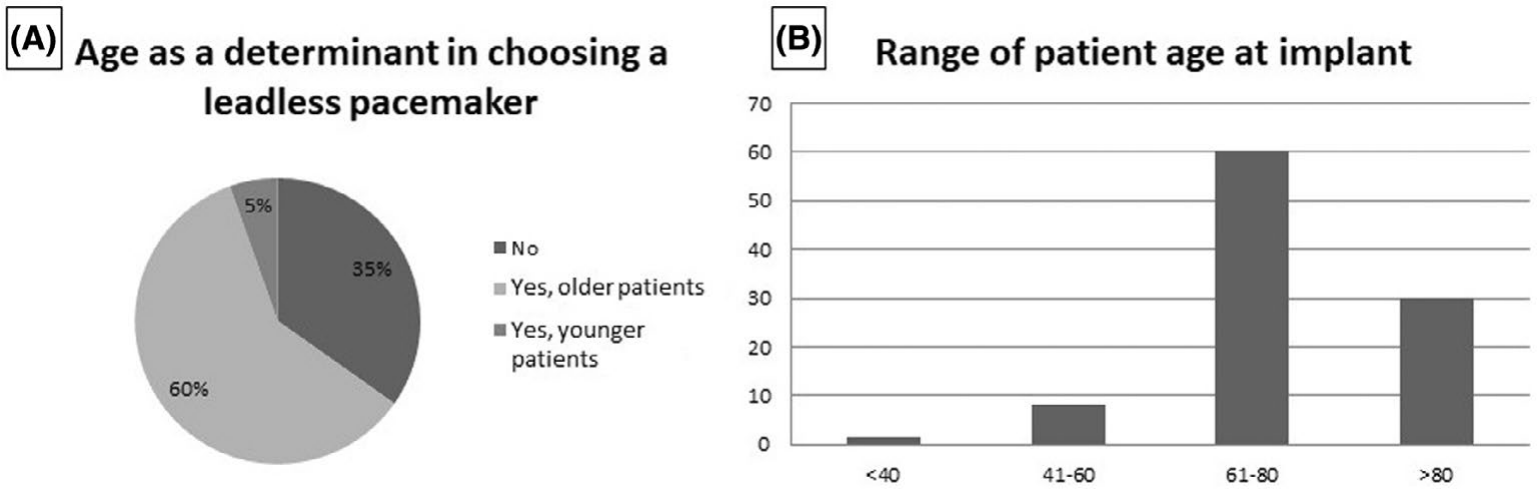
Age as a determinant factor in choosing a leadless pacemaker instead of a transvenous (A) and age range of candidates for leadless pacemaker implant (B). In B, the vertical axis reports the percentage of the center and the horizontal axis the age range.

As shown in Figure 3A, the age of the patient candidate for the L‐PM implant was considered a determinant by 65% of the responders, while the remaining 35% considered age not important. In fact, 60% of the responders considered older patients more suitable candidates for the lower probability of device replacement during their life, and the remaining 5% preferred younger patients based on the assumption that the L‐PM could be a more acceptable option for them. As to the age range at implant, 60% of the responding centers considered age between 61 and 80 years the most suitable, 30% age above 80 years, 8% age between 40 and 60 years, and only 1% age below 40 years (Figure 3B). Nineteen participants preferred not to respond on the age range of candidates, as they did not implant L‐PMs.

Most of the responders (56%) never implanted or would never implant a L‐PM in a patient requiring a dual‐chamber PM. For 32% of the responders, this was considered acceptable in less than 10% of the cases, and for 12% of responders, in 10%–50% of these patients.

### 3.3 Limitations to leadless pacemaker implant and barriers to device adoption

**FIGURE 4:**
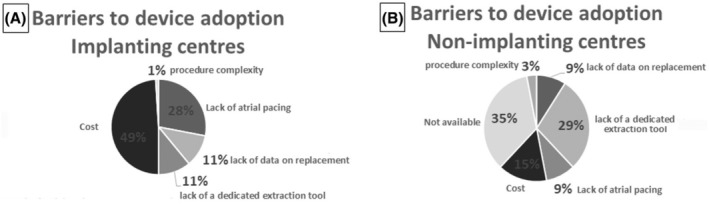
Barriers to device adoption in implanting (A) and non‐implanting (B) centers.

The participants were then asked to identify the factors limiting the implant of a L‐PM in the implanting centers and the barriers to L‐PM adoption in non‐implanting centers. In the first group (Figure 4A), the main reason was the cost of the device, accounting for 49% of the responses, followed by lack of atrial pacing (28%), lack of a dedicated extraction tool (11%), lack of data on replacement (11%), and procedure complexity (1%). For the participants not implanting a L‐PM (Figure 4B), the main barrier to the use of this technology was that the device was not made available at their site (35%), followed by lack of a dedicated extraction tool (29%), cost (15%), lack of atrial pacing (9%), lack of data on how to manage device replacement (9%), and procedure complexity (3%). Of interest, among the non‐implanting centers, 38% were high‐volume centers (>200 PM implanted de novo yearly) and 34% had an intermediate volume of PM implants (101–200 yearly).

### 3.4 Organizational aspects for leadless pacemaker implantation

**FIGURE 5:**
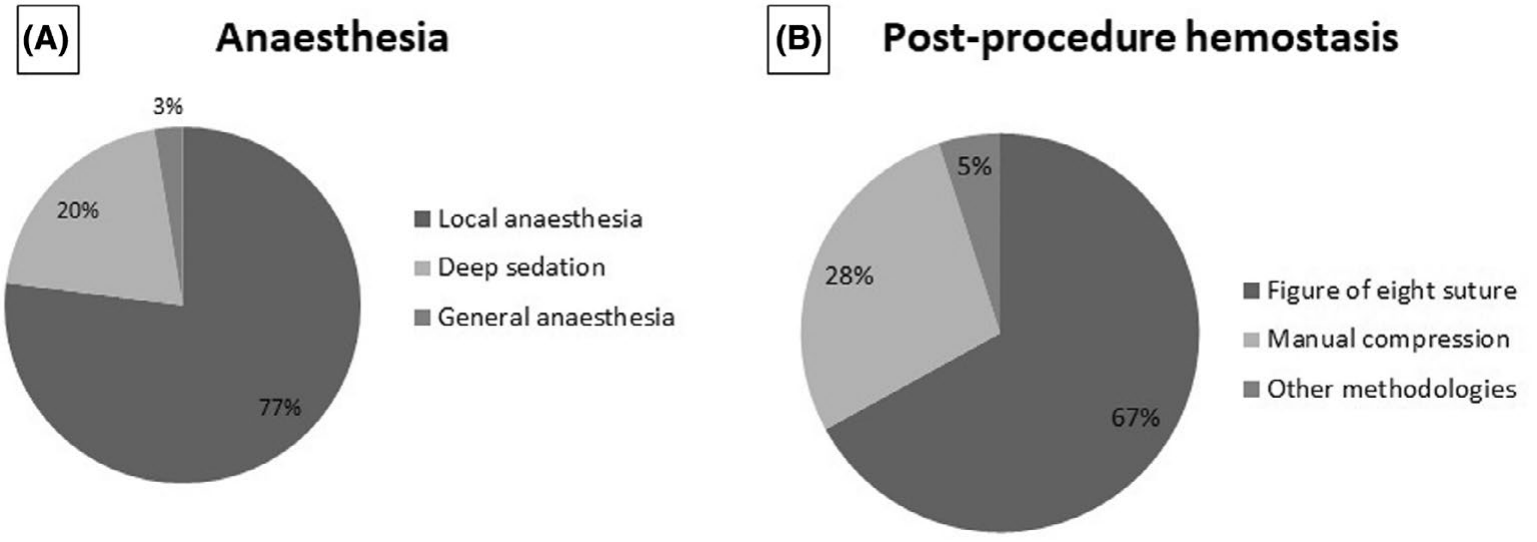
Type of anesthesia chosen by the implanting centers (A) and type of hemostasis of the vascular access (B).

As shown in Figure 5A, in 77% of the implanting centers, the L‐PM implant was performed only with local anaesthesia; in 20%, deep sedation was used, and in only 3% of the centers, the procedure was performed under general anaesthesia. Most of the centers (67%) reported using the figure of eight suture as the standard haemostatic method at the end of the procedure; only manual compression was used in 28% of the centers, and other methodologies were reported in the remaining 5% of the centers (Figure 5B).

**FIGURE 6:**
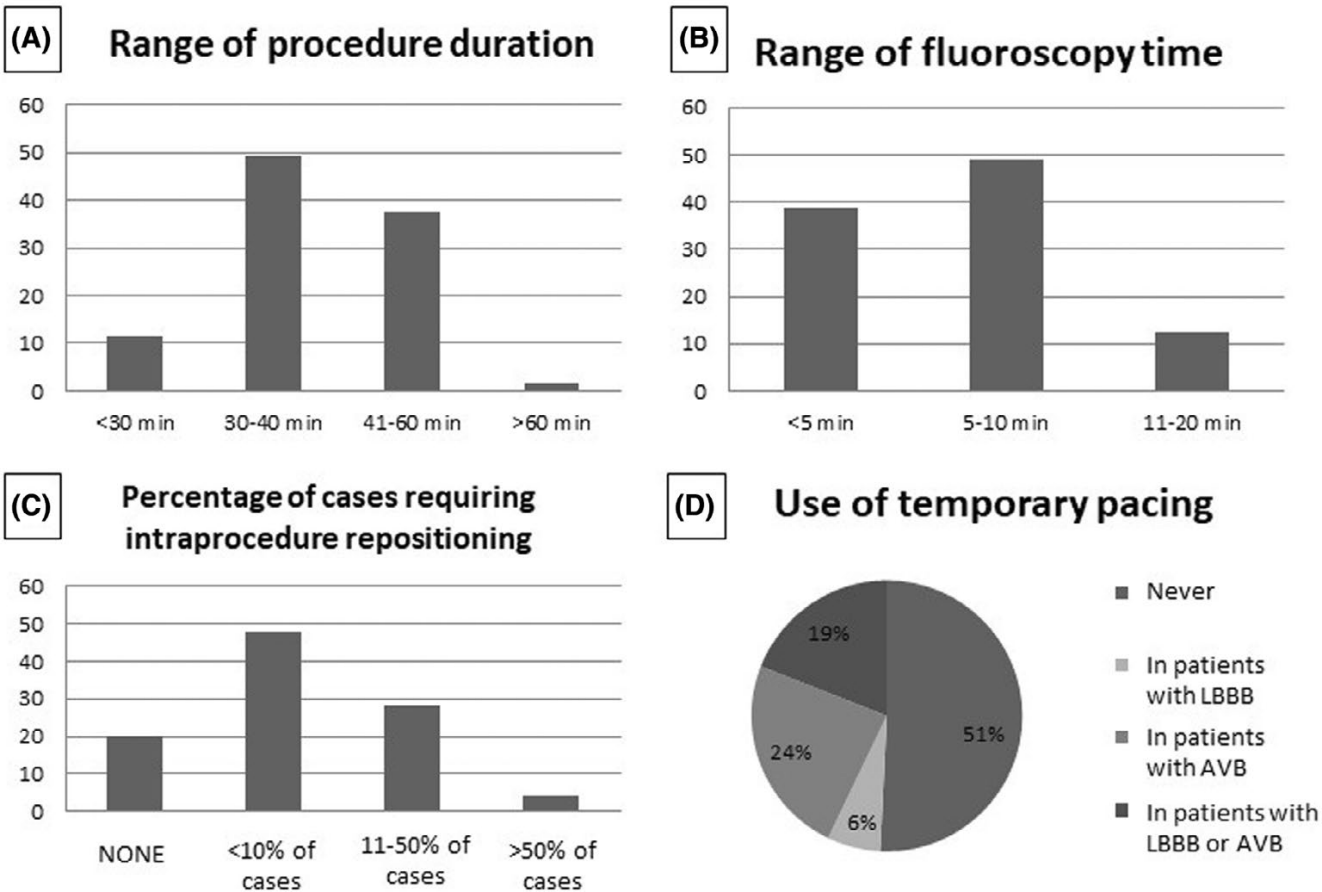
Intraprocedure variables reported by the implanting centers in terms of average procedure duration (A), fluoroscopy time (B), need for intraprocedure repositioning (C), and use of temporary pacing (D). In A, B, and C, the vertical axis indicates the percentage of implanting centers, while the horizontal axis indicates the range of the considered variable. AVB, atrioventricular block; LBBB, left bundle branch block.

In general, the L‐PM implant was associated with a short procedure duration, lasting longer than 60 min only in 1 center. A mean procedure time shorter than 40 min was reported by the majority of the centers (Figure 6A). Similarly, fluoroscopy time was reported to be within 10 min by 88% of the responders (Figure 6B). Interestingly, among the 33 centers with a low (up to 10) annual volume of L‐PM implants, all but 1 reported a procedure time within 60 min, and all but three reported a fluoroscopy time within 10 min. As to the need for intraprocedure device repositioning (i.e., device positioning requiring more than one attempt) in the cases performed over the previous year, most of the responders (48%) reported that this occurred in less than 10% of the procedures, while 28% reported the need for repositioning in 11%–50% of the procedures (Figure 6C). About half of the implanting centers (51%) had never positioned a catheter for temporary pacing during an L‐PM implant, while in the remaining centers, patients presenting with atrioventricular block and/or patients with left bundle branch block received temporary pacing (Figure 6E).

Forty‐two implanting centers (67%) reported no complications, while the remaining 21 (33%) reported complications. The most frequently reported complication was related to the vascular access, reported by 13 centers, and in four of them only manual compression for haemostasis was used. Interestingly, the percentage of centers reporting complications, calculated on the number of centers with a similar L‐PM implant volume, showed a trend to increase according to the center volume. In fact, regardless of the severity of complication, centers reporting complications accounted for only 27% of the centers implanting 1–10 L‐PM yearly, 33% of those implanting 11–20 L‐PM, and increased to 45% of those implanting >20 L‐PM. Severe complications such as cardiac tamponade were reported only by 2 centers, one implanting 1–10 L‐PM and the other implanting 11–20 L‐PM.

### 3.5 Post‐implant follow‐up

**FIGURE 7:**
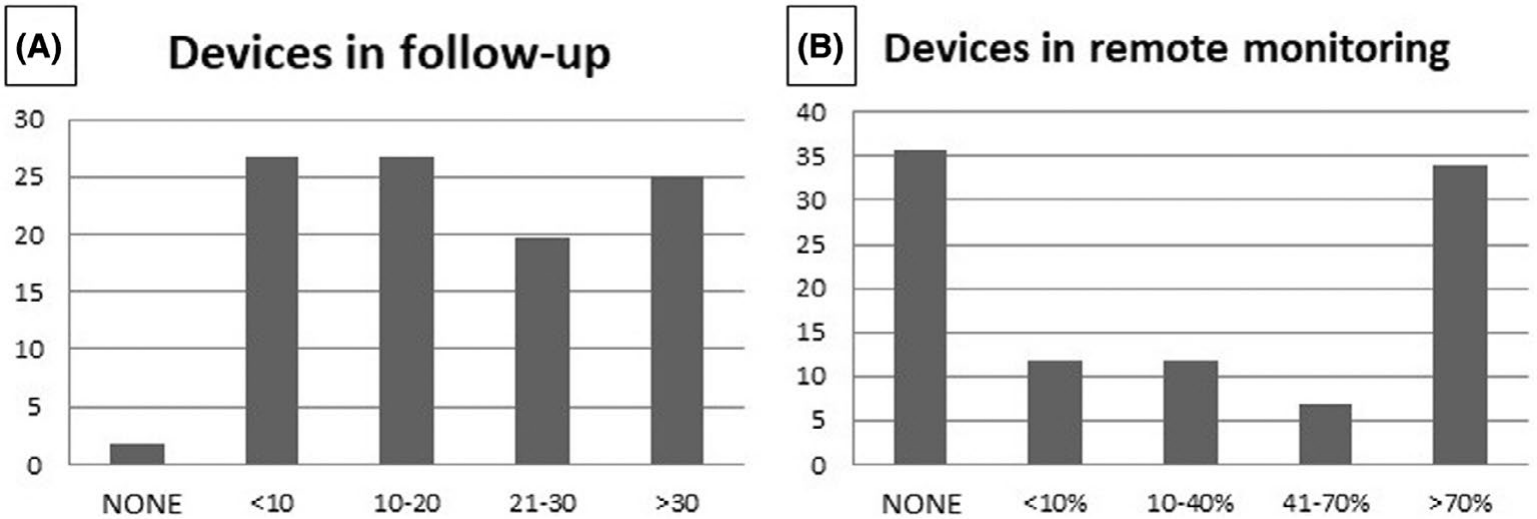
Follow‐up data in terms of the number of patients with a leadless pacemaker in follow‐up (A) and use of remote monitoring (B). The vertical axis indicates the percentage of implanting centers, while the horizontal axis indicates the number of devices in follow‐up.

As shown in Figure 7A, 44% of the responders reported that a relatively high number of patients implanted with a L‐PM were in follow‐up in their center: more than 30 patients in 25% of the centers and between 21 and 30 in 19%. Remote monitoring was variably used in different centers: 34% of the responders followed up remotely >70% of the implanted patients, whereas another 35% preferred in‐office follow‐up (Figure 7B). The remaining centers used remote monitoring in a variable percentage of implanted patients. During follow‐up, in only five implanting centers (8%) malfunction of a single L‐PM was observed with the need to switch to another pacing modality; among these centers, only one was a low‐volume center for L‐PM implantation.

## 4 DISCUSSION

The present study reports the results of an Italian survey that aims to evaluate how L‐PM is utilized and perceived in everyday clinical practice. The main strength of this survey is that both centers implanting and not implanting L‐PM were included, thus giving more insights into what the factors are that indeed limit a wider use of this technology. Moreover, this survey included both high, intermediate, and low‐volume centers, giving a real‐life picture of everyday clinical practice of L‐PM implant in Italy.

The implant of a L‐PM is still reserved for a minority of patients as assessed by the fact that most of the participating centers reported less than 20 L‐PM implants per year. Although the reasons for limiting the adoption of this technology in Italy seem various, the main barrier to a wider use of L‐PM among both implanting and non‐implanting centers seems related to device cost, addressed by roughly 50% of the responders in both groups. In fact, in the latter group, the unavailability of the L‐PM is very likely related to the device cost as most non‐implanting physicians, who accounted for 33% of the responders, were from high‐ or intermediate‐volume centers with the potential of having a non‐negligible number of candidates for L‐PM implant. This, in turn, might discourage hospital administrations from making this technology available, as the costs may significantly rise. This issue should be specifically considered in light that Italy has a universal tax‐based healthcare system, and the device is available at an average cost of €7000. This is approximately 3.5 times higher than a transvenous single‐chamber pacemaker and there is no dedicated reimbursement for this new technology.

Our findings are in agreement with previous surveys conducted among European centers in 2018 and 2020 that showed a low adoption of L‐PM implantation in everyday clinical practice.^17^, ^18^ Although L‐PM has been introduced in 2015 and the number of implanting centers has progressively increased, no significant change in the penetrance of the therapy and in the factors limiting a wider use of this technology has been observed over time.

The main factors limiting a larger penetration of this technology into clinical practice are the cost of the device, the lack of atrial pacing and of a dedicated tool for L‐PM retrieval, and the issue of how to deal with L‐PM replacement at the time of battery exhaustion. With this in mind, it is not surprising that L‐PM implantation is mostly reserved for patients with anatomic contraindications to transvenous PM implantation, higher infective risk profiles and/or history of prior transvenous PM extraction for infection. Moreover, most responders reported that L‐PM implant is more likely to be chosen for patients older than 60 years of age. Specifically, about one third of the responders reported that the age of candidates for a L‐PM was over 80 years, and only one center declared having implanted patients below 40 years of age. In our and previous surveys, age emerged as a critical issue.^17^, ^18^, ^19^ Theoretically, considering the expected lower risk of lead and endovascular complications with L‐PM in a long‐term follow‐up, younger patients should be the ideal candidates. However, concerns about how to manage battery replacement and the possible future need for device upgrade limit its use in this group of patients, favoring the elderly.

Another important issue is the lack of atrial pacing in L‐PMs available at the time of the survey. Actually, the majority of the responders reported that they had never implanted or would never implant a L‐PM in patients with an indication for a dual‐chamber PM. Also, the lack of dedicated tools for lead extraction was reported as an important limiting factor. Both problems might be addressed in the near future by the introduction into clinical practice of a dual‐chamber L‐PM^14^ and of newer technologies.^20^, ^21^ Meanwhile, it is not surprising that L‐PM is still confined to a minority of patients, as those with an expected higher infective risk profile.^9^, ^22^, ^23^, ^24^

Concerning the L‐PM implant procedure, the results of this survey are in agreement with previous reports from European centers on procedure duration and sedation management.^25^ Indeed, L‐PM implant was reported to be a straightforward procedure as assessed by mean duration below 40 min in the majority of the cases, short fluoroscopy duration, and need of multiple deployment attempts on few occasions. Moreover, the use of temporary pacing was limited to selected cases of patients with complete atrioventricular block or pre‐existing left bundle branch block. Local anaesthesia and deep sedation were chosen in most of the cases, suggesting that implant of L‐PM is a well‐tolerated procedure.

Complications were reported by about one third of the participants and, not surprisingly, complications related to vascular access were the most frequently reported. Cardiac tamponade, although rare, was also observed as previously reported in other studies.^25^, ^26^, ^27^ The use of the septal implant site confirmed by contrast dye injection under high‐resolution fluoroscopy should be preferred, and the personnel should be trained and equipped to deal with the potential occurrence of cardiac perforation and tamponade.^28^ The lower percentage of centers reporting complications among the low‐volume centers could lead to a hypothesis that L‐PM implantation can be safely performed even at the beginning of the learning curve and in low‐volume centers. On the other hand, a higher percentage of centers reporting complications among the high‐volume centers might be related to patient‐specific anatomic variables encountered more frequently as the number of treated cases increases and to wider patient selection criteria adopted in high‐volume centers. Indeed, these centers are likely to select more complex cases for these procedures, which could justify a higher percentage of centers reporting complications among those with a higher volume of L‐PM implants.

## 5 LIMITATIONS

This survey has some intrinsic limitations. First, the number of proposed questions was limited; therefore, not covering all aspects related to L‐PM implant. The participating centers accounted for 44.5% of censored Italian arrhythmia centers. For this reason, our findings should be interpreted with caution, as they could not accurately reflect in detail the Italian clinical practice. All questions of the questionnaire were multiple‐choice questions. This type of questionnaire is time‐efficient, and responses are easy to code and interpret. On the other hand, the surveys based on multiple‐choice questions have some limitations. Responders are required to choose a response that might not exactly reflect their answer. In addition, the subjective design of questionnaires and multiple‐choice questions with preconceived categories may not fully allow a detailed representation of clinical reality. Data on complications and malfunctions were analyzed in an aggregate way, as the exact number of complications and of L‐PM implants was not individually asked for each center, as it can be the aim of an observational multicenter study, which is beyond the aim of this survey. For the same reason, we did not collect the clinical characteristics of patients implanted at each center, precluding an in‐depth analysis of the adverse events according to the clinical variables. Finally, the survey was based on voluntary participation, thus possibly creating an unintended selection bias.

## 6 CONCLUSIONS

This survey provides a contemporary insight on the use of L‐PM across nationwide implantation centres. Although L‐PM implantation has become available since a decade ago and its use has increased progressively, there are still many factors limiting a wider penetration of this therapy into everyday clinical practice. In this Italian experience, the costs, the absence of atrial pacing, and of a dedicated tool for device retrieval, together with concern on how to replace the L‐PM, still limit implants to a selected group of patients. New technologies, becoming available in the near future, might change the current scenario.

## CONFLICT OF INTEREST STATEMENT

RR Received modest speaking fees from Abbott and Boston Scientific; MC received honoraria for lectures from Biosense Webster and Abbott; GP received honoraria for lectures from Biotronik; VR received honoraria for lectures from Medtronic; RDP received honoraria for lectures and scientific collaboration from Johnson & Johnson Medical and Medtronic. The other authors have nothing to declare.

## Supporting information


Data S1.

## Data Availability

Complete data related to the present work will be made available by the corresponding author upon reasonable request.

## APPENDIX A

List of the participants according to regions. CALABRIA: Giovanni Bisignani, Ospedale Civile Ferrari, Castrovillari (CS), Antonio Pangallo Azienda Ospedaliera Bianchi‐Melacrino‐Morelli, Reggio Calabria, Gianluca Quirino UOC Cardiologia, Azienda Ospedaliera di Cosenza, Cosenza, Antonio Strangio, Ospedale Civile “San Giovanni di Dio”, Crotone; CAMPANIA: Antonio D'Onofrio AORN “Dei Colli”—Ospedale Monaldi—UOSD Elettrofisiologia Studio e Terapia delle Aritmie, Napoli, Felice Nappi, AORN Moscati, Avellino, Antonio Rapacciuolo, Dipartimento di Scienze Biomediche Avanzate, Università degli Studi di Napoli Federico II, Napoli, Vincenzo Russo Unità Sincope Università della Campania “Luigi Vanvitelli”—Ospedale Monaldi, Napoli, Giuseppe Stabile Clinica San Michele Maddaloni; EMILIA ROMAGNA: Cristina Balla and Matteo Bertini Azienda Ospedaliero‐Universitaria S. Anna, Ferrara, Paolo Pastori Ospedale di Fidenza, Fidenza (PR), Angelo Placci, Azienda Ospedaliero‐Universitaria, Parma; Davide Saporito Ospedale Infermi—ASL Romagna, Rimini, Matteo Ziacchi Istituto di Cardiologia, IRCCS Azienda Ospedaliero Universitaria di Bologna, Bologna; LAZIO: Sergio Calcagno Ospedale San Paolo, Civittavecchia (RM), Marco De Giusti, Azienda Ospedaliera San Giovanni Addolorata, Roma, Ermenegildo De Ruvo Policlinico Casilino, Roma, Maria Chiara Gatto INMI IRCCS Lazzaro Spallanzani, Roma, Carlo Lavalle Policlinico Umberto I—Università “Sapienza” di Roma, Roma, Francesco Messina Azienda Ospedaliera S. Camillo‐Forlanini, Roma, Nicolino Patruno Ospedale Dei Castelli, Ariccia (RM), Gemma Pelargonio IRCCS Fondazione Policlinico Agostino Gemelli, Roma, Renato Pietro Ricci Centro Cardio‐Aritmologico, Roma, Luca Santini UOC Cardiologia Ospedale G.B. Grassi, Roma; LIGURIA: Francesco Pentimalli Ospedale S. Paolo, Savona; LOMBARDIA: Patrizia Albonico ASST Sette Laghi Ospedale Galmarini, Tradate (VA), Giuseppina Belotti ASST Bergamo Ovest Ospedale di Treviglio (BG), Manuela Bianchi ASST PAVIA Ospedale Civile di Vigevano, Vigevano (PV), Enrico Chieffo ASST Crema Ospedale Maggiore di Crema, Crema (CR), Roberto De Ponti, Lorenzo Adriano Doni ASST Settelaghi, Ospedale di Circolo‐Università dell'Insubria, Varese, Giovanni Battista Forleo Ospedale Sacco, Milano, Patrizio Mazzone Ospedale Niguarda, Milano, Maria Silvia Negroni Ospedale Civile di Vigevano, Vigevano (PV), Antonio Pani ASST Lecco Ospedale Manzoni, Lecco, Patrizia Pepi ASST Mantova Ospedale Carlo Poma, Mantova, Giovanni Battista Perego Istituto Auxologico Italiano—Ospedale S. Luca, Milano, Amedeo Prezioso Fondazione Poliambulanza Istituto Ospedaliero, Brescia, Roberto Rordorf UOS Aritmologia ed. Elettrofisiologia IRCCS Policlinico S. Matteo, Pavia, Luigi Vasaturo Ospedale Predabissi, Vizzolo Predabissi (MI), Domanico Zagari Istituto Clinico Humanitas Mater Domini Castellanza (VA); MARCHE: Laura Cipolletta and Federico Guerra Azienda Ospedaliero‐Universitaria delle Marche, Ancona; MOLISE: Matteo Santamaria Fondazione Agostino Gemelli, Campobasso; PIEMONTE: Claudia Amellone Ospedale Maria Vittoria, Torino, Gabriele Dell'Era Azienda Ospedaliero‐Universitaria Ospedale Maggiore della Carità, Novara, Aldo Coppolino Ospedale SS. Annunziata, Savigliano (CN), Elisa Ebrille Ospedale Maria Vittoria, Torino, Federico Ferraris, Mario Matta Città della Salute e della Scienza, Torino, Ilaria Meynet Ospedale degli Infermi, Rivoli (TO), Umberto Parravicini Ospedale SS. Trinità Borgomanero (NO), Fabrizio Pizzetti Ospedale Santo Spirito, Casale Monferrato (AL); PUGLIA: Michele Accogli, Giovanni Coluccia, Pietro Palmisano Azienda Ospedaliera Cardinale Giovanni Panico, Tricase (LE), Cosimo Damiano Dicandia Anthea Hospital, Bari, Maurelio Domanico Lauretti, Ennio Pisanò Azienda Ospedaliera Vito Fazzi, Lecce, Zefferino Palamà, Mariano Rillo Casa di Cura Villa Verde, Taranto, Pier Luigi Pellegrino Azienda Ospedaliero‐Universitaria Policlinico Riuniti, Foggia, Rafel Sai Presidio Ospedaliero Sacro Cuore di Gesù, Gallipoli (LE), Pietro Scicchitano Presidio Ospedaliero F. Perinei Altamura (BA), Federica Troisi Ospedale Generale Regionale F. Miulli, Acquaviva delle Fonti (BA), Pierpaolo Vitti Ospedale Bonomo, Andria (BA); SARDEGNA: Roberto Floris Ospedale Nostra Signora di Bonaria, S. Gavino Monreale (CA), Vincenzo Nissardi Policlinico D. Casula Monserrato (CA); SICILIA: Gianfranco Ciaramitaro, Giuseppe Coppola, Alessandro Faraci Policlinico P. Giaccone, Palermo, Michele Di Silvestro Ospedale Umberto I, Enna, Marco Salvatore Maria Lisi Azienda Ospedaliera Cannizzaro, Catania, Antonino Mignano Presidio Ospedaliero Villa Sofia, Palermo, Giuseppe Sgarito ARNAS Ospedale Civico, Palermo; TOSCANA: Giuseppe Arena Cardiologia USL Nordovest Toscana, Massa Carrara, Marzia Giaccardi, Alessandro Paoletti Pierini Ospedale S. Maria Nuova, Firenze, Luca Panchetti, Marcello Piacenti, Andrea Rossi Fondazione Toscana Gabriele Monasterio, Pisa; TRENTINO ALTO ADIGE: Massimiliano Maines Ospedale S: Maria del Carmine, Rovereto (TN); Chiara Andreoli Ospedale S: Giovanni Battista, Foligno (PG); VENETO: Emanuele Bertaglia Ospedale di Camposampiero, Camposampiero (PD), Mauro Fantinel Ospedale di Feltre, Feltre (BL), Lina Marcantoni Ospedale S. Maria della Misericordia, Rovigo, Federico Migliore Azienda Ospedaliero‐Universitaria di Padova, Padova, Giulio Molon Ospedale Sacro Cuore Don Calabria, Negrar (VR), Nicola Padovani Ospedale Mater Salutis, Legnago (VR), Antonio Rossillo Ospedale S. Bortolo, Vicenza.
