# Supplementary material for: Leadless pacemaker implantation in real‐world clinical practice: An Italian survey promoted by the AIAC (Italian Association of Arrhythmology and Cardiac Pacing)
Source: J Arrhythm. 2025 Mar 24;41(2):e70045. doi: 10.1002/joa3.70045 (PMC11931594; doi:10.1002/joa3.70045)
Supplement: Supplementary file 1 — Data S1. [file JOA3-41-e70045-s001.docx]

**Questionnaire of the survey on leadless pacemaker**

1. How many new pacemaker implants (single-chamber, dual-chamber, biventricular, leadless) are performed annually in your center?

- <50
- 50-100
- 101-200
- >200

1. How many new single-chamber pacemaker implants are performed annually in your center?

- <30
- 30-50
- 51-100
- >100

1. How many new dual-chamber pacemaker implants are performed annually in your center?

- <30
- 30-50
- 51-100
- >100

1. How many new biventricular devices implants (CRT-D or CRT-P) are performed annually in your center?

- <30
- 30-50
- 51-100
- >100

1. How many leadless pacemaker implants are performed annually in your center?

- none
- 1-10
- 11-20
- 21-30
- >30

1. Indicate the three main reasons that in the last year led you (or could have led you if you usually do not implant leadless pacemaker) to choose a leadless pacemaker over a conventional pacemaker? (multiple choice possible up to a maximum of 3 answers)

- Contraindication to traditional pacemaker implantation for anatomic reasons
- Permanent atrial fibrillation
- Patient’s high risk of infections
- Previous extraction of devices due to infection
- Previous extraction of devices due to malfunction
- 1^st^ or 2^nd^ degree AV block
- Patient characteristics
- Physician’s choice
- Patient’s choice

1. Is age a factor that significantly influences (or could influence if you do not implant) the choice of whether to implant a leadless pacemaker?

- Yes, it is preferably used in elderly patients who will be less likely to have to undergo multiple replacements during follow-up
- Yes, it is preferably offered to young patients to encourage acceptance of the implant
- No, it is not a determining factor

1. What is the average age of patients in whom you have implanted, or you would implant a leadless pacemaker in your centre?

- <40 years old
- 40-60 years old
- 61-80 years old
- >80 years old

1. What is the main factor limiting the use of leadless pacemakers in your center?

- Costs
- Lack of atrial pacing
- Lack of a dedicated system for extraction/retrieval of the device in the event of malfunction
- Complexity of the implanting procedure
- Uncertainty regarding the management of replacement
- The device is not available in my hospital
- My center is not authorized to perform this procedure

END OF THE SURVEY FOPR THOSE ANSWERING “NONE” AT QUESTION N°5

1. In how many cases did you choose a single-chamber leadless pacemaker even though the patient would have been indicated for a dual-chamber pacemaker?

- Never
- <10% of patients
- 10-50% of patients
- >50% of patients

1. What type of anesthesia do you most frequently use for leadless pacemaker implants?

- Local
- Deep sedation
- General anesthesia

1. What is the average duration of the leadless pacemaker implant procedure (skin-to-skin) in your center?

- <30 min
- 30-40 min
- 41-60 min
- >60 min

1. What is the average fluoroscopy time during a leadless pacemaker implant in your center?

- <5 min
- 5-10 min
- 11-20 min
- >20 min

1. Over the last year, what was the percentage of leadless pacemaker implant procedures in which more than one attempt at device positioning (release) was necessary to obtain a satisfactory position?

- Never
- <10%
- 11-50%
- >50%
- In all implants

1. What hemostatic method is used at the site of femoral vein access?

- Manual compression
- “Figure-of-eight” suture
- Other

1. In which cases do you use a temporary pacemaker during the leadless pacemaker implant procedure?

- Never
- In case of AV block
- When LBBB is present
- In case of AV block or LBBB
- Always

1. What complications have occurred during leadless pacemaker implants in your center? (multiple choice possible)

- None
- Complications in the vascular access site
- Pericardial effusion
- Cardiac tamponade
- Displacement of the device
- Death
- Capture/sensing defect

1. How many leadless devices are in follow-up in your center?

- <10
- 10-20
- 21-30
- >30
- none

1. In how many cases was malfunction observed?

- none
- 1
- 1-5
- >5

1. In how many cases a switch to a transvenous single-chamber device was necessary?

- none
- 1
- 1-5
- >5

1. In how many cases a switch to a transvenous dual-chamber or CRT device was necessary?

- none
- 1
- 1-5
- >5

1. How many patients with a leadless PM in follow-up in your center have remote monitoring?

- none
- <10%
- 10-40%
- 41-70%
- >70%
